# Partial dehiscence of an intraoperative staple line during laparoscopic sleeve gastrectomy: A case report

**DOI:** 10.1016/j.ijscr.2021.106642

**Published:** 2021-11-30

**Authors:** Abdullah A. Albarrak

**Affiliations:** Department of Surgery, College of Medicine, Majmaah University, Industrial area, Al Majma'ah 15341, Saudi Arabia

**Keywords:** Sleeve gastrectomy, Leakage, Stapler, Case report

## Abstract

**Introduction:**

Leakage along a staple line during sleeve gastrectomy is a serious complication. Mechanical causes are uncommon; however, they should be considered as sources of acute postoperative leaks. The presented case discusses an important intraoperative complication with an avoidable cause that could benefit practicing surgeons as well as residents in training programs.

**Presentation of case:**

This case describes the mechanical failure of a stapler that was identified intraoperatively. The staple line was oversewn using a 3–0 V Lok suture. The methylene blue test was negative, and the patient had an uneventful postoperative recovery.

**Discussion:**

While most leaks were attributed to ischemia of the upper third of the stomach, leaks occurring within the first three postoperative days have a different pathophysiology. This may involve mechanical complications (stapler failure), direct gastric tissue trauma from aggressive handling, or thermal injuries. In our case, the likely cause of the partial dehiscence was the proximity of the stapler to the bougie and an unnoticed small fold at the antrum.

**Conclusion:**

Surgeons should avoid placing the stapler too close to the bougie. Furthermore, surgeons should ensure that the stomach is flat and there are no gastric folds that could lead to stapling failure.

## Highlights

• Staple line dehiscence in sleeve gastrectomy is a serious complication.
• We recommend separating the stapler and bougie and avoiding gastric wall folds.
• Oversewing the staple line was sufficient to address dehiscence in this case.

## 1 Introduction

Laparoscopic sleeve gastrectomy is the most common procedure performed on morbidly obese patients worldwide owing to its low risk of morbidity. It is also a straightforward technical procedure with satisfactory weight loss outcomes [1]. In addition, laparoscopic sleeve gastrectomy is shown to resolve hypertension in 62.17% and improve hypertension control in 35.7% after a mean of 5 years in a systematic review [2]. The procedure involves dissecting the greater omentum from the greater curvature of the stomach, the greater curvature is freed from the antrum till the angle of Hiss exposing the left crus by dividing the gastrocolic (greater omentum), gastrosplenic, and gastophrenic ligaments followed by creating a sleeve tube over a bougie based on the lesser curvature using surgical staplers during the reconstruction of the stomach [3].

Leakage along the staple line is a serious complication of sleeve gastrectomy. It has a reported prevalence of 1–3%, if sleeve construction is performed as a primary procedure [4]. There are multiple theories of sleeve leakage. It may develop due to ischemia of the upper third of the stomach that typically manifests five to six days postoperatively. However, earlier leakage occurs due to mechanical failure of the stapler or direct gastric trauma from intraoperative handling of the stomach.

This paper reports a case of partial dehiscence of a staple line during laparoscopic sleeve gastrectomy. We also review the management of dehiscence and patient outcomes, and discuss possible causes of this complication and how to prevent it.

All the parts of the manuscript are in line with the SCARE criteria [5].

## 2 Presentation of case

A 41-year-old male patient presented to the clinic. He had morbid central obesity with a body mass index of 43, height 173 cm and weight 130 kg. The patient had a history of successful treatment for hepatitis C virus (negative polymerase chain reaction result after therapy) and hypertension, which was controlled by medications. He had no other reported medical comorbidities or history of surgical procedures. The patient denied any gastrointestinal symptoms prior to surgery. The systematic review of this patient was normal, and he consented and electively booked for laparoscopic sleeve gastrectomy.

The author, a certified bariatric surgeon, performed the procedure. The surgery started by using Veress needle insufflation, followed by optical trocar entry and secondary placement of the four ports. The greater omentum was released, and the fundus was dissected exposing the medial edge of the left crus.

**Fig. 1:**
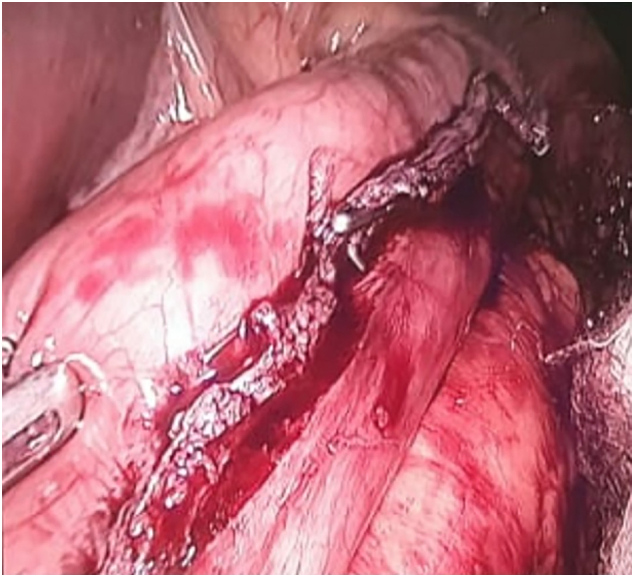
Partial dehiscence of the staple line.

Prior to stapling, a 36-F bougie was inserted into the stomach and positioned such that its tip was located in the distal gastric antrum. The stapler was an Ethicon Echelon Flex GST 60 (Ethicon San Angelo, TX, USA), which was black with an open staple height of 4.2 mm, and a closed staple height of 2.3 mm. The stapler was placed such that the antral transection began 4 cm proximal to the pylorus. The stapler was closed for 30 s prior to staple discharge. After completion, the staple line was inspected, and partial dehiscence was observed, as shown in Fig. 1.

A second black cartridge was used to staple around the incisura, resulting in the formation of a perfect staple line. The third and fourth staples were discharged from the green cartridge, and the final staple was discharged from the blue cartridge. The staple line was checked for hemostasis, and clips were placed at all the sources of bleeding.

**Fig. 2:**
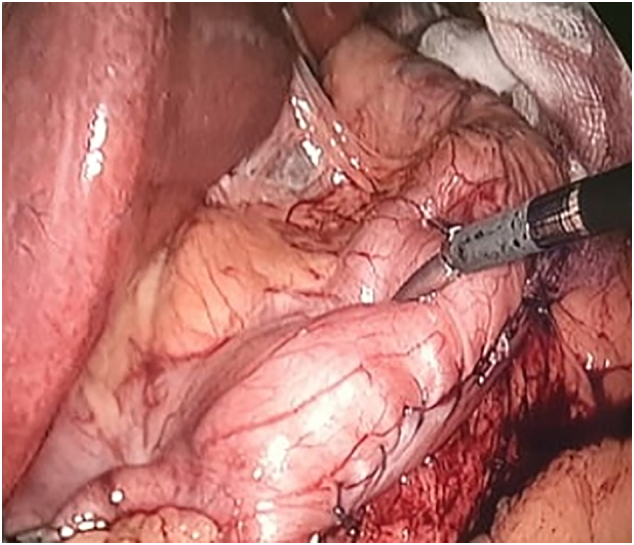
Methylene blue test using a bougie in the proximal stomach. (For interpretation of the references to colour in this figure legend, the reader is referred to the web version of this article.)

The affected staple line was oversewed at the dehiscence site with a barbed, V-Loc™ 180 suture and intact bougie. This was carefully done to prevent the stomach from twisting or narrowing. Upon completion, the bougie was pulled back to the upper stomach, and 60-cc of methylene blue was used to test for leakage with gastric outlet occlusion, as shown in Fig. 2. There was no apparent leak, and the stomach size was appropriate with no twisting or obstruction. The procedure was terminated after the stomach had been removed and the right midclavicular line port fascia was closed. No drain was inserted since the closure was sufficient.

The patient's postoperative recovery was uneventful. Unfortunately, fluoroscopy was unavailable due to challenges regarding maintenance. The patient remained in the hospital for 3 days, and this procedure was performed on the last day of the week. The patient's pain was adequately managed with intravenous acetaminophen (1 g every 6 h), and his vital signs remained normal throughout the duration of hospitalization. The patient was allowed to drink clear liquids on day 2 and was discharged on day 3 with normal vitals, adequate tolerance to clear fluids, and a normal abdominal examination and white cell count. The patient was given discharge instructions and instructed to return to the hospital if he experienced increasing abdominal pain, fever, fatigue, or liquid intolerance.

Four months postoperatively, the patient has remained healthy, stopped his antihypertensive medication, and has lost nearly 50% of his excess body weight; his weight was 98 kg. This indicates a complication classification of Clavien-Dindo I. The patient was happy about his experience with the surgery and his outcome.

## 3 Discussion

Sleeve leakage is a serious complication of laparoscopic sleeve gastrectomy. Leaks are classified based on their timing: acute (within seven days), early (between one and five weeks), late (between six weeks and three months), and chronic (> three months). Most leaks occur after five to seven postoperative days [6]. While most leaks are attributed to ischemia of the upper third of the stomach, leaks occurring within the first three postoperative days have a different pathophysiology. This may involve mechanical complications (stapler failure), direct gastric tissue trauma from aggressive handling, or thermal injuries [4].

Based on the surgical video of our case, the likely cause of the partial dehiscence was the proximity of the stapler to the bougie and an unnoticed small fold at the antrum. Other causes were less likely because the discharge of the staple was preceded by sufficient compression time, and the cartridge had a closed staple height of 2.3 mm. Staple line reinforcement would not prevent this since the buttressing material took a part of the closed staple height that was already compromised by the antral fold.

Oversewing the staple line sufficiently addressed the dehiscence in this case. However, some surgeons may choose not to address the partial dehiscence at all.

We reviewed the literature on the discharge of the first staple. One reported method involved placing the bougie after the first staple had been discharged into the antrum [7]. However, this approach was not recommended in other studies [6], [8].

## 4 Conclusion

Based on our experience with this case, we recommend placing the bougie away from the stapler to prevent folds in the antrum caused by the stapler's jaw.

## Data availability

Patient's data and photographs are available with the author.

## Funding

This research did not receive any specific grant from funding agencies in the public, commercial, or not-for-profit sectors.

## Ethical approval

The ethical committee approved the writing and publication of this case report.

The writing and publication of this work was approved by the ethical committee of the surgery department, King Khalid Majmaah Hospital.

## Consent

Written informed consent was obtained from the patient for publication of this case report and accompanying images. A copy of the written consent is available for review by the Editor-in-Chief of this journal on request.

The patient provided written informed consent for the case report and the use and publication of photos of his surgery.

## Guarantor

The author, Dr. Abdullah A Albarrak.

## Registration of research studies

N/A.

## Provenance and peer review

Not commissioned, externally peer-reviewed.

## CRediT authorship contribution statement

Not applicable.

## Declaration of competing interest

The author declares no conflict of interest.

## References

[1] Angrisani L., Santonicola A., Iovino P., Vitiello A., Zundel N., Buchwald H. (2017). Bariatric surgery and endoluminal procedures: IFSO worldwide survey 2014. Obes. Surg..

[2] Graham C., Switzer N., Reso A., Armstrong C., Church N., Mitchell P. (2019). Sleeve gastrectomy and hypertension: a systematic review of long-term outcomes. Surg. Endosc..

[3] Bhandari M., Fobi M.A.L., Buchwald J.N. (2019). Standardization of bariatric metabolic procedures: world consensus meeting statement. Obes. Surg..

[4] Abou Rached A., Basile M., El Masri H. (2014). Gastric leaks post sleeve gastrectomy: review of its prevention and management. World J. Gastroenterol..

[5] Agha R.A., Franchi T., Sohrabi C., Mathew G., Kerwan A., SCARE Group (2020). The SCARE 2020 guideline: updating consensus Surgical Case Report (SCARE) guidelines. Int. J. Surg..

[6] Rosenthal R.J., Diaz A.A., Arvidsson D., Baker R.S., Basso N., Bellanger D., International Sleeve Gastrectomy Expert Panel (2012). International Sleeve Gastrectomy Expert Panel Consensus Statement: best practice guidelines based on experience of >12,000 cases. Surg. Obes. Relat. Dis..

[7] Ramos A.C., Bastos E.L., Ramos M.G., Bertin N.T., Galvão T.D., de Lucena R.T. (2015). Technical aspects of laparoscopic sleeve gastrectomy. Arq. Bras. Cir. Dig..

[8] Palermo M., Cardoso A.R., Gagner M., Gagner M., Cardoso A.R., Palermo M., Noel P., Nocca D. (2020). The Perfect Sleeve Gastrectomy: A Clinical Guide to Evaluation, Treatment, and Techniques.

